# Efficacy of Ingesting an Oral Rehydration Solution after Exercise on Fluid Balance and Endurance Performance

**DOI:** 10.3390/nu12123826

**Published:** 2020-12-15

**Authors:** Priscilla Weiping Fan, Stephen F. Burns, Jason Kai Wei Lee

**Affiliations:** 1 DSO National Laboratories, Defence Medical and Environmental Research Institute, Singapore S117510, Singapore; fweiping@dso.org.sg; 2 Physical Education and Sports Science, National Institute of Education, Nanyang Technological University, Singapore S637616, Singapore; stephen.burns@nie.edu.sg; 3 Human Potential Translational Research Programme, Yong Loo Lin School of Medicine, National University of Singapore, Singapore S117597, Singapore; 4 Department of Physiology, Yong Loo Lin School of Medicine, National University of Singapore, Singapore S117593, Singapore; 5 Global Asia Institute, National University of Singapore, Singapore S119076, Singapore; 6 N.1 Institute for Health, National University of Singapore, Singapore S117456, Singapore; 7 Institute for Digital Medicine, National University of Singapore, Singapore S117456, Singapore

**Keywords:** fluid retention, palatability, endurance exercise performance, heat, sodium

## Abstract

This study investigated the efficacy of ingesting an oral rehydration solution (DD) that has a high electrolyte concentration after exercise on fluid balance and cycling performance in comparison with a sports drink (SD) and water (WA). Nine healthy males aged 24 ± 2 years (mean ± SD), with peak oxygen uptake (VO_2_ peak) 55 ± 6 mL·kg^−1^·min^−1^ completed three experimental trials in a randomised manner ingesting WA, SD (carbohydrates: 62 g·L^−1^, sodium: 31 ± 3 mmol·L^−1^) or DD (carbohydrates: 33 g·L^−1^, sodium: 60 ± 3 mmol·L^−1^). On all trials, fluid was ingested during 75 min cycling at 65% VO_2_ peak (temperature: 30.4 ± 0.3 °C, relative humidity: 76 ± 1%, simulated wind speed: 8.0 ± 0.6 m·s^−1^) and during 2 h of recovery (temperature: 23.0 ± 1.0 °C, relative humidity: 67 ± 2%), with the total volume equivalent to 150% of sweat loss during the ride. A 45 min pre-load cycling time trial at a 65% VO_2_ peak followed by a 20 km time trial was conducted after a further 3 h of recovery. Fluid retention was higher with DD (30 ± 15%) than WA (−4 ± 19%; *p* < 0.001) and SD (10 ± 15%; *p* = 0.002). Mean ratings of palatability were similar among drinks (WA: 4.25 ± 2.60; SD: 5.61 ± 1.79; DD: 5.40 ± 1.58; *p* = 0.33). Although time trial performance was similar across all three trials (WA: 2365 ± 321 s; SD: 2252 ± 174 s; DD: 2268 ± 184 s; *p* = 0.65), the completion time was faster in eight participants with SD and seven participants with DD than with WA. Comparing SD with DD, completion time was reduced in five participants and increased in four participants. DD was more effective at restoring the fluid deficit during recovery from exercise than SD and WA without compromising the drink’s palatability with increased sodium concentration. Most individuals demonstrated better endurance exercise time trial performance with DD and SD than with WA.

## 1. Introduction

The debilitating effects of exercise-induced dehydration on exercise performance are well documented [1,2]. Conversely, exercise-associated hyponatraemia (EAH) is a potentially fatal condition largely resulting from excessive fluid intake beyond the capacity for fluid excretion and/or the lack of available solution to replace the sodium lost during exercise [3,4].

Commercial carbohydrate–electrolyte sports drinks are formulated to replace the fluid and electrolytes lost through sweat, promote voluntary fluid consumption, and improve net water absorption [5]. Several studies have investigated the effects of ingesting commercially available sports drinks on the restoration of fluid balance after exercise-induced fluid deficits and found that they were generally more effective in rehydration than water or other placebo solutions [6,7,8] as a lower volume of urine was produced [9]. The higher sodium content of sports drinks might be a key factor for improved rehydration as increased sodium concentration in beverages has been shown to blunt urine output and promote more effective rehydration [6,7,9,10]. Apart from urine volume, the effects of sports drinks on key thermal parameters such as core temperature (*T_c_*) and skin temperature (*T_sk_*) can also affect endurance performance [11]. Moreover, sports drinks have been shown to augment endurance capacity in subsequent exercise bouts completed on the same day as the initial fluid loss [7].

The volume of urine produced in a healthy individual is largely determined by circulating hormones, in particular, by levels of anti-diuretic hormone (ADH) and aldosterone. ADH, also known as arginine vasopressin, plays a key role in homeostasis, and the regulation of water, glucose, and salts in the blood [12]. It is released when the body is dehydrated and increases with decreasing plasma volume and increasing plasma osmolality, such as during or after prolonged physical exercise [13]. This causes the kidneys to conserve water, thus concentrating the urine and reducing urine volume [14]. Aldosterone, on the other hand, regulates electrolyte levels, i.e., sodium and potassium [15]. It increases during salt deprivation, dehydration, heat exposure and psychosocial stress. A reduction in the stimulus for aldosterone release, together with reduced plasma renin activity, has been linked to the increased urinary loss of sodium and water [16]. Investigating these two hormones may ascertain the underlying mechanisms responsible for improved fluid retention after accruing exercise-induced fluid deficits.

As most commercially available sports drinks typically contain a small amount of sodium (5–30 mmol·L^−1^; [5]), they only partially replace the sodium lost through sweating in humans during exercise (40–60 mmol·L^−1^; [17]). The main limitation behind increasing sodium in drinks relates to poor palatability at higher concentrations which could lead to a lower consumption volume if a drink is provided ad libitum [9]. The solution to be used as a post-exercise recovery drink in the present study, an oral rehydration solution, has reportedly overcome this barrier by increasing sodium concentrations without compromising palatability and compensates for the average sodium sweat loss in humans during exercise. The higher concentration of electrolytes in the rehydration solutions can, in theory, enhance the replacement of electrolytes lost during exercise. This same solution was previously compared with a commercially available sports drink and fluid retention was found to be similar between the test solutions [18]. It is noteworthy that the 30 min duration catered for post-exercise rehydration in that study might not be sufficient for the test solutions to be fully absorbed for fluid retention to be accurately assessed. While oral rehydration solution has been investigated for its effect on fluid balance [18,19], its applied impact on a functional outcome such as time trial performance is unknown. Thus, this study aimed to investigate the rehydration efficacy of ingesting an oral rehydration solution during post-exercise recovery and on subsequent pre-load time trial performance.

## 2. Materials and Methods

### 2.1. Participants

This study was approved by the local Institutional Review Board (Reference No.: DSO DMERI/1-5). Nine physically active males (mean ± standard deviation (SD); age 24 ± 2 years, body mass 63.0 ± 7.5 kg, height 1.76 ± 0.06 cm, body mass index (BMI) 20.3 ± 1.8 kg·m^−2^, body surface area (BSA) 1.77 ± 0.12 m^2^, body fat 11.4 ± 3.0%, VO_2_ peak 55 ± 6 mL·kg^−1^·min^−1^, peak power output 286 ± 42 W) were recruited to participate in the study. All participants took part in physical activities/sports (including cycling) for at least five sessions a week. Informed consent was given in writing after reading the participant information sheet describing the nature, benefits, and risks of the study before any study procedures began.

### 2.2. Experimental Design

Participants completed five visits to the testing laboratory: (i) anthropometric measurements and peak oxygen uptake testing; (ii) familiarisation trial; (iii) experimental trial with water (WA); (iv) experimental trial with a carbohydrate–electrolyte sports drink (SD); and (v) an experimental trial with an oral rehydration solution (DD). The three experimental trials (iii–v) were conducted in a randomised order using a Latin Square design to minimise any order effects. Each trial was separated by a minimum of 6 days and a maximum of 15 days to allow adequate recovery and minimise any training effects. Treatments were administered in a crossover manner, with both researchers and volunteers blinded to the provision of the SD and DD i.e., the participants were informed that the aim of this study was to investigate different rehydration solutions during exercise and recovery on the subsequent pre-load time trial performance. This would also minimise any psychological assumption that the rehydration solutions were likely to augment their cycling performance. It should be noted that the WA trial was not blinded to participants.

### 2.3. Anthropometric and Peak Oxygen Uptake Measurements

On the first visit to the laboratory, each participant had his nude body mass measured to the nearest 0.001 kg using a precision weighing scale (IND690/KCC150, Mettler Toledo, Albstadt, Germany). Height was obtained to the nearest 0.5 cm using a stadiometer (Seca, Brooklyn, NY, USA). BMI was calculated as (body mass in kg)**/**(height in m)^2^. BSA was estimated using the equation of Du Bois [20]. Skinfold thickness measured in mm was taken at four sites (biceps, triceps, subscapular and suprailiac) in triplicate using skinfold callipers (HSK-BI, British Indicators, West Sussex, UK). The mean value at each site was used to calculate the total skinfold thickness. Body density was calculated according to the estimation of Durnin and Womersley [21] with the percent body fat estimated using the equation of Siri [22].

Peak oxygen uptake (VO_2_ peak) and power output were obtained via a continuous incremental test on a cycle ergometer (Velotron Pro, RacerMate, Seattle, USA). The test commenced at a workload of 105 W, with increments of 35 W every 3 min until volitional exhaustion. Expired air samples, heart rate (monitored by short-range telemetry; S810i, Polar Electro Oy, Kempele, Finland) and the ratings of perceived exertion (RPE; [23]) were collected during the final min of each stage. Expired air was monitored via a metabolic cart (TrueOne 2400^®^, ParvoMedics, Sandy, UT, USA). Based on the relationship between peak oxygen uptake and power output, the power output equivalent to 65% VO_2_ peak was calculated for use during the subsequent familiarisation and experimental trials.

### 2.4. Familiarisation

The familiarisation trial consisted of a 75 min ride followed by a 45 min pre-load time trial and a 20 km time trial. This mirrored the protocol of the experimental trials to ensure each participant was accustomed to the test setting, instrumentation and demands. This also allowed the verification of the required power output of 65% VO_2_ peak for the experimental trials. Water was ingested during this trial.

### 2.5. Control of Pre-Trial Status

Participants were asked to standardise their dietary intake 48 h prior to the commencement of each experimental trial to minimise the differences in the metabolic and hydration status among trials. The dietary intake 48 h prior to the first experimental trial was recorded and repeated for the remaining trials (48–24 h: total energy 2192 ± 888 kcal, protein 100 ± 55 g, fat 89 ± 65 g, carbohydrates 253 ± 48 g; 24–0 h: total energy 2285 ± 767 kcal, protein 89 ± 34 g, fat 73 ± 39 g, carbohydrates 319 ± 118 g). They were also requested to avoid strenuous activity and to refrain from alcohol 24 h prior to each trial. A diet and physical activity record sheet was kept to facilitate compliance with the requirements. To enhance ecological validity and decrease the likelihood that the participants began each trial with a pre-existing energy and fluid deficit, they were asked to eat a standardised breakfast comprising four slices of wholemeal bread with hazelnut cocoa spread (Nutella, Ferrero SpA, Alba, Italy) and consume a ready-to-drink pack of chocolate malted dairy milk (Milo, Nestlé S.A., Vevey, Switzerland; total energy: 878 kcal, protein: 19 g, fat: 37 g, carbohydrate: 117 g). In addition, the participants were asked to drink 500 mL of water before arriving at the testing laboratory. The ingestion of breakfast and water was done at least 60 min prior to the commencement of the trials.

### 2.6. Experimental Trials

Participants performed three experimental trials ingesting WA, SD (100Plus, Fraser and Neave, Limited, Singapore; carbohydrate = 6.2%) and DD (Drip Drop, Inc., San Francisco, CA, USA; carbohydrate = 3.3%; Table 1). The experimental trials commenced in the morning at the same time to control for circadian variations in *T_c_* [24].

Upon arrival at the laboratory, a mid-stream urine sample was collected. A cannula was inserted into a superficial vein on the dorsal surface of the forearm. Nude body mass was recorded to the nearest 0.001 kg. A telemetric check was performed using an ambulatory *T_c_* data recording system (VitalSense^®^, Mini Mitter Co., Inc., Bend, OR, USA) to ensure a *T_c_* sensor ingested between 8 and 10 h prior to the trial was residing within the volunteer and transmitting a signal. The ambulatory *T_c_* data-recording device was placed in a sealed waterproof bag, fitted into a padded pouch, and worn on a customised lightweight harness around the waist, with the data recorder positioned in the lumbar region. A chest band and a wrist-watch heart rate monitor (S810i, Polar Electro Oy, Kempele, Finland) were secured onto each volunteer for heart rate (HR) measurement. Four telemetric iButtons (Maxim Integrated Products, Inc., Sunnyvale, CA, USA) were placed on the participants’ right upper chest, right triceps, right anterior thigh and right calf to monitor *T_sk_*. The iButtons were secured with a waterproof medical dressing (Tegaderm™ Flim, 3 M Health Care, St Paul, MN, USA). Similar hydration status before each trial was indicated by the consistency of body mass, Hgb concentration, Hct, and serum and urine osmolality

Participants entered an environmental chamber in which the ambient temperature was set at 30 °C with relative humidity of 75% and wind velocity of ~8.0 m/s. Environmental conditions were measured by a climatic logger (Squirrel, 2020 Series, Grant Instruments, Cambridge, England).

Baseline measurements of *T_c_* and HR were obtained every 5 min for 20 min with the participant seated on a chair in the chamber. After 10 min of seated rest, the participant’s hand and forearm was immersed in warm water for a further 10 min to promote the arterialisation of the venous blood [25] before a venous blood sample (8 mL) was withdrawn. Following the resting blood sampling, the volunteers began cycling at 65% VO_2_ peak for 75 min. Gastrointestinal temperature and HR were recorded every 5 min during the 75 min ride at 65% VO_2_ peak. Expired air was collected over a 2-min period every 30 min. The ratings of perceived exertion, thermal sensation (TS; [26]) and thirst using a 10-centimetre visual analogue scale (0 = not at all, 10 = very) were recorded every 15 min during exercise. Fluid equivalent to 1.5 mL·kg^−1^ body mass was provided immediately every 15 min. A fluid questionnaire was completed after ingestion of each fluid bolus during exercise and recovery. Utilising 10 cm visual analogue scales (0 = not at all, 10 = very), the questionnaire assessed subjective responses to sweetness, saltiness and palatability, and associated feelings of thirst quenching and mouth taste [7].

Upon the completion of the 75 min cycle ride, a second 8 mL venous blood sample was taken. Participants exited the chamber and provided a full urine sample. Body mass was measured within 10 min after the end of exercise following the removal of any unevaporated sweat with a towel. Immediately after blood sampling, the participants were seated for the next 5 h except for toilet breaks. In the first two hours, they were provided with equal portions of one of the test drinks every 15 min. The total volume of test drink ingested was equivalent to 150% of the fluid lost as sweat (inclusive of the volume ingested during the preceding 75 min ride at 65% VO_2_ peak). Sweat loss was estimated from the differences in body mass, corrected for fluid intake and urine production before the start and at the end of exercise, and not corrected for respiratory water loss and metabolic water production. Any urine produced during recovery was accumulated as the volume at the end of each hour. One kilogram of body mass loss was assumed to be one litre of sweat loss. Percent fluid retention was calculated using Equation (1):

Percent fluid retention (%) = [(Total fluid ingested (mL) − Urine output (mL)/Total fluid ingested (mL)] × 100%.(1)

Further blood samples were taken 1, 2, 3 and 5 h into recovery and a urine sample at each hour for 5 h. After the final blood and urine samples at 5 h of recovery, the participants were weighed nude. They changed into shorts and cycling cleats and donned a heart rate monitor and ambulatory *T_c_* data-recording device. Participants then completed a pre-load time trial which consisted of a 45 min cycle at 65% VO_2_ peak followed by a 20 km time trial. Pre-load time trials were used as a measure of endurance performance [27,28]. Environmental conditions were the same as the preceding 75 min ride. *T_c_* and HR were recorded every 5 min. The ratings of perceived exertion and TS were recorded at 15 min intervals during the pre-load and at every 2 km during the time trial. Portions of fluid equivalent to 1.5 mL·kg^−1^ body mass were provided every 15 min during the pre-load time trial and at every 5 km during the time trial. Time trial performance was recorded, but this information was withheld from the participants until they have completed the study. A full urine sample was collected, and nude body mass was measured within 10 min at the end of exercise following the removal of any unevaporated sweat with a towel.

### 2.7. Blood Sampling and Analysis

Venous blood samples (8 mL each) were taken after the participants were seated for at least 10 min to minimise postural effects on circulating blood volume [29]. For each 8 mL of blood sampled, approximately 3 mL was dispensed into a tube containing K_2_EDTA while the remaining blood was dispensed into a tube containing no anticoagulant and stored temporarily in ice until the end of each trial.

Before pipetting the samples for analysis, blood in tubes containing K_2-_ ethylenediaminetetraacetic acid (EDTA) were gently mixed on a roller mixer for at least 15 min. The EDTA-treated blood was used to obtain spun haematocrit (Hct) and determine haemoglobin (Hgb) concentration by an automatic haematology analyser (ACT diff 2, Beckman Coulter, Miami, FL, USA). The relative changes in plasma, blood and red cell volumes during exercise were calculated from the Hgb concentrations and Hct values were obtained with reference to the resting sample [30]. Blood glucose was measured from whole blood using a portable hand-held analyser (Acc-Chek^®^ Advantage, Roche, Mannheim, Germany). The remaining EDTA-treated blood was centrifuged at 4 °C and 3500 rpm for 10 min (BR4i, Jouan, Saint-Herblain, France) to allow for the extraction of plasma. The plasma was stored at −80 °C for the determination of an ADH concentration using a radioimmunoassay (RIA; BÜHLMANN Laboratories AG, Schönenbuch, Switzerland) at a later point.

The tube containing no anticoagulant was left to stand for 30 min before the serum was separated in the refrigerated centrifuge. The serum obtained was refrigerated and used for the determination of sodium, potassium and osmolality. Serum osmolality was determined by freezing point depression (Osmomat 030, Gonotec, YSI, Farnborough, UK). Serum sodium and potassium concentrations were determined via an electrolyte analyser (AVL 9181 Electrolyte Analyzer, AVL Scientific Corporation, Roswell, GA, USA). The remaining serum was stored at −80 °C for the determination of aldosterone concentration using RIA (BÜHLMANN Laboratories AG, Switzerland). Duplicate measurements were made for all blood parameters except for Hct which was measured in triplicate.

### 2.8. Urine and Drink Analysis

The urine sample collected from volunteers for each trial would typically represent the second bladder void of the day. A full urine volume was collected using a plastic measuring cylinder before, during and after each trial, and samples were stored in 10-millilitre containers with the remaining volume discarded. The volume of urine output was measured and the samples were analysed for osmolality. A drink sample from each trial was analysed for osmolality, sodium and potassium concentrations (Table 1).

There were no differences in the physiological parameters measured prior to the experimental trials (Table 2). Participants exhibited a similar hydration status before each trial, indicated by the consistency in body mass, Hgb concentration, Hct, and serum and urine osmolality (all *p* > 0.05).

### 2.9. Statistical Analyses

All statistical computations were performed using the Statistical Package for Social Sciences version 15.0. The normality of data was assessed using the Shapiro–Wilk test. A one-factor ANOVA was performed to evaluate the differences among the trials in the measured variables at a single time point and a two-factor (i.e., trial and time) repeated measures ANOVA evaluated the changes in the remaining measured variables over time (the number of time points computed was in accordance with the reported sampling intervals described earlier). Time trial performance was analysed up to 30 min and at completion to include all nine participants. One-factor ANOVA was employed to isolate the differences among treatment means. For all statistical analyses, the 0.05 level of significance was used. The effect size (Hedges’ g) and 95% confidence intervals of the rehydration efficacy and time trial performance were calculated as a qualitative measure of the strength of the differences among rehydration drinks. This was derived using the mean differences in variables divided by the pooled standard deviation. The magnitude of g was classified according to the scale by Hopkins and colleagues [31]: 0–0.2 = trivial; 0.2–0.6 = small; 0.6–1.2 = moderate; 1.2–2.0 = large; 2.0–4.0 = very large; >4.0 = extremely large. Data are presented as the mean ± SD and figures are illustrated as the mean ± standard error of the mean (SE).

## 3. Results

One participant experienced difficulty in ingesting a volume equivalent to 150% of his fluid lost in the first experimental trial and therefore was provided with a total volume of 120% of fluid lost in all experimental trials. *T_c_* was reported for seven participants during the first bout of the 75 min exercise as readings for two participants were affected by fluid ingestion indicated by sudden sharp dips upon fluid ingestion. Blood data were reported for eight participants as the blood collected from one participant lysed. During the 20 km time trial, data were reported up to 30 min because it was the final time point that all participants reached.

### 3.1. Environmental Conditions

There were no differences in the environmental conditions during the 75 min cycle at 65% VO_2_ peak (ambient temperature: WA 30.3 ± 0.2 °C vs. SD 30.5 ± 0.2 °C vs. DD 30.5 ± 0.3 °C, *p* = 0.19; relative humidity: WA 76 ± 1% vs. SD 76 ± 1% vs. DD 75 ± 1%, *p* = 0.87; wind velocity: WA 8.0 ± 0.6 m·s^−1^ vs. SD 8.2 ± 0.7 m·s^−1^ vs. DD 8.0 ± 0.6 m·s^−1^, *p* = 0.78) and pre-load cycling time trial (ambient temperature: WA 30.3 ± 0.2 °C vs. SD 30.4 ± 0.2 °C vs. DD 30.4 ± 0.2 °C, *p* = 0.50; relative humidity: WA 76 ± 1% vs. SD 75 ± 1% vs. DD 76 ± 1%, *p* = 0.11; wind velocity: 8.0 ± 0.7 m·s^−1^ vs. SD 8.1 ± 0.5 m·s^−1^ vs. DD 8.0 ± 1.0 m·s^−1^, *p* = 0.71). The ambient temperature was ~23 °C with 67% relative humidity during the 5 h recovery. 

### 3.2. Core (Gastrointestinal) Temperature

There were no differences in the mean *T_c_* during the first 75 min of cycling (WA: 37.6 ± 0.2 °C vs. SD: 37.7 ± 0.1 °C vs. DD: 37.7 ± 0.2 °C; *p* = 0.85), the second bout of 45 min pre-load exercise (WA: 37.7 ± 0.3 °C vs. SD: 37.7 ± 0.2 °C vs. DD: 37.6 ± 0.3 °C; *p* = 0.69) and subsequent 20 km time trial (WA: 38.1 ± 0.3 °C vs. SD: 38.1 ± 0.3 °C vs. DD: 38.2 ± 0.2 °C; *p* = 0.57). *T_c_* at completion of 20 km time trial was higher with DD than WA (WA: 38.2 ± 0.3 °C vs. DD: 38.5 ± 0.2 °C; *p* = 0.008).

### 3.3. Skin Temperature

Mean *T_sk_* were similar among trials during the first 75 min exercise bout (WA: 33.2 ± 0.5 °C vs. SD: 33.5 ± 0.4 °C vs. DD: 33.4 ± 0.7 °C; *p* = 0.45), second bout of 45 min pre-load exercise (WA: 33.2 ± 0.3 °C vs. SD: 33.5 ± 0.4 °C vs. DD: 33.4 ± 0.7 °C; *p* = 0.58 and subsequent 20 km time trial (WA: 33.1 ± 0.4 °C vs. SD: 33.6 ± 0.5 °C vs. DD: 33.3 ± 0.9 °C; *p* = 0.33). There were no differences in the *T_sk_* across trials at the completion of a 20 km time trial (WA: 32.7 ± 0.7 °C vs. SD: 33.2 ± 0.7 °C vs. DD: 33.1 ± 1.1 °C; *p* = 0.40).

### 3.4. Heart Rate

The mean HR did not differ among trials during the first 75 min exercise bout (WA: 130 ± 9 b·min^−1^ vs. SD: 133 ± 8 b·min^−1^ vs. DD: 133 ± 9 b·min^−1^; *p* = 0.77), second 45 min pre-load exercise (WA: 136 ± 10 b·min^−1^ vs. SD: 134 ± 8 b·min^−1^ vs. DD: 135 ± 7 b·min^−1^; *p* = 0.90) and subsequent 20 km time trial (WA: 142 ± 12 b·min^−1^ vs. SD: 145 ± 9 b·min^−1^ vs. DD: 144 ± 9 b·min^−1^; *p* = 0.79). HR was similar across trials upon completion of the 20 km time trial (WA: 159 ± 18 b·min^−1^ vs. SD: 165 ± 13 b·min^−1^ vs. DD: 161 ± 10 b·min^−1^; *p* = 0.61).

### 3.5. Fluid Balance

**Figure 1:**
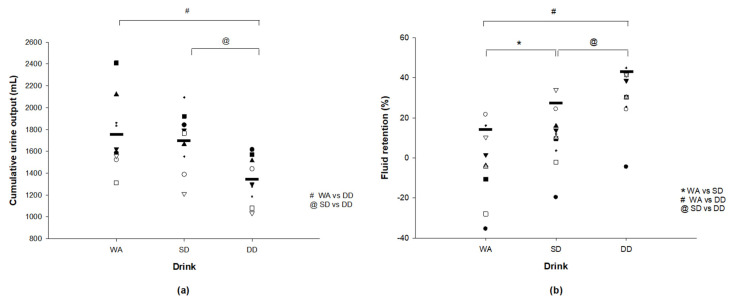
(**a**) Individual cumulative urine output and (**b**) individual percent fluid retention during 5 h of recovery. * SD significantly different from WA (*p* < 0.05). ^#^ DD significantly different from SD (*p* < 0.05). ^@^ DD significantly different from SD (*p* < 0.05). “-” depicts the mean value for (**a**) Cumulative urine output (WA: 1759 ± 338 mL, SD: 1693 ± 273 mL, DD: 1335 ± 212 mL); and (**b**) fluid retention (WA: 16 ± 13%, SD: 25 ± 12%, DD: 42 ± 12%).

Sweat loss was similar during the first 75 min exercise bout (WA: 1201 ± 279 mL vs. SD: 1320 ± 231 mL vs. DD: 1348 ± 241 mL; *p* = 0.43) and therefore the total fluid intake was similar among trials (WA: 1734 ± 414 mL vs. SD: 1901 ± 266 mL vs. DD: 1945 ± 281 mL; *p* = 0.37). Cumulative urine output over the 5 h of recovery was lower with DD (1335 ± 212 mL) than with WA (1759 ± 338 mL; *p* = 0.002) and SD (1693 ± 273 mL; *p* = 0.004; Figure 1a). A small effect size of 0.20 (−1.09, 0.68) was observed between the WA and SD while a large effect size was observed between the WA and DD (1.43 (0.43, 2.42)), and between the SD and DD (1.39 (0.40, 2.38)). Similarly, fluid retention was higher with DD (30 ± 15%) than with WA (−4 ± 19%; *p* < 0.001) and SD (10 ± 15%; *p* = 0.002; Figure 1b). Ingesting SD resulted in a higher fluid retention than with ingesting WA (*p* = 0.002). A moderate effect size of 0.78 (−0.14, 1.69) was found between WA and SD while a large effect size was found between the WA and DD (1.89 (0.82, 2.97)), and between SD and DD (1.27 (0.30, 2.24)). However, net fluid balance remained negative at the end of the 5 h period with no differences found across trials (WA: −2.4 ± 0.7% vs. SD: −2.0 ± 0.7% vs. DD: −1.3 ± 0.8%; *p* = 0.20). The effect size was small between WA and SD (0.54 (−0.35, 1.44)), large between WA and DD (1.39 (0.40, 2.38)), and moderate between SD and DD (0.89 (−0.04, 1.81)).

### 3.6. Ratings of Perceived Exertion, Thermal Sensation and Thirst

No differences were observed in the mean ratings of perceived exertion across trials during the 75 min cycling (WA: 11 ± 1 vs. SD: 12 ± 1 vs. DD: 12 ± 1; *p* = 0.53), the 45 min pre-load cycling (WA: 12 ± 1 vs. SD: 12 ± 1 vs. DD: 12 ± 1, *p* = 0.79) and the 20 km time trial (WA: 14 ± 1 vs. SD: 15 ± 1 vs. DD: 14 ± 1; *p* = 0.71). Mean ratings of TS were similar across trials between the 75 min cycling (5 ± 1; *p* = 0.63), 45 min pre-load cycling (5 ± 1; *p* = 0.99) and 20 km time trial (5 ± 1; *p* = 0.92). There were no differences in mean ratings of thirst across the trials during the 75 min cycling (WA: 5.16 ± 1.49 vs. SD: 5.09 ± 1.52 vs. DD: 5.04 ± 1.50; *p* = 0.98) and 45 min pre-load cycling (WA: 5.60 ± 1.42 vs. SD: 4.74 ± 1.72 vs. DD: 4.95 ± 1.64; *p* = 0.50).

### 3.7. Subjective Ratings of Drinks

SD (6.24 ± 1.68) and DD (5.26 ± 2.22) were rated sweeter than WA (0.32 ± 0.68; *p* = 0.008) while similar ratings of sweetness were found between SD and DD (*p* = 0.12). Mean ratings of saltiness were higher when ingesting SD (3.54 ± 2.45; *p* = 0.015) and DD (4.94 ± 2.48; *p* = 0.008) than with WA (0.31 ± 0.66) while ratings of saltiness were similar between SD and DD (*p* = 0.02). Mean ratings of palatability were similar among all drinks (WA: 4.25 ± 2.60 vs. SD: 5.61 ± 1.79 vs. DD: 5.40 ± 1.58; *p* = 0.33). Compared with WA (4.20 ± 1.59), ratings of thirst quenching were higher with SD (5.98 ± 1.34; *p* = 0.006) and DD (5.71 ± 1.31; *p* = 0.008). Ratings of thirst quenching were similar between SD and DD (*p* = 0.20). Mouth taste was rated higher when ingesting SD (5.60 ± 1.76) and DD (5.82 ± 2.31) than with WA (1.38 ± 1.84; *p* = 0.008) but the mouth taste was similar between SD and DD (*p* = 0.86).

### 3.8. Plasma Volume

**Figure 2:**
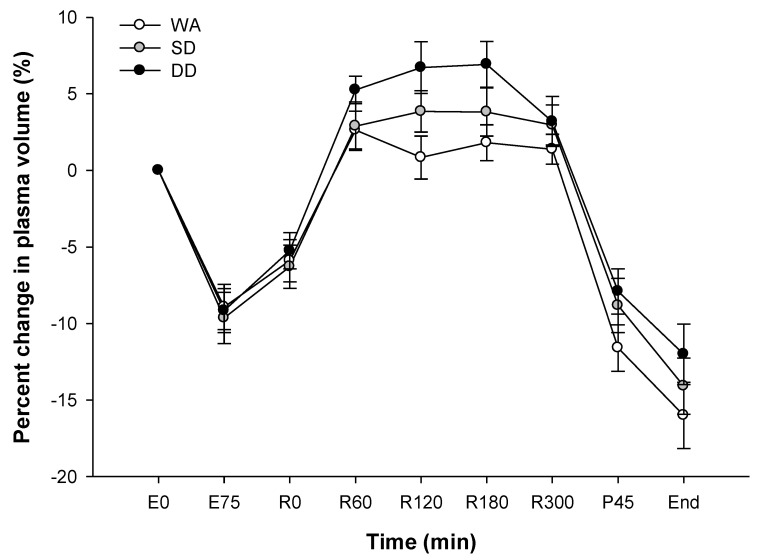
Percent change in the plasma volume during the experimental trials. First bout of 75 min cycle: E0 and E75; 5 h recovery: R0–R300; end of pre-load: P45; completion of 20 km time trial: end. Values are the mean ± SE.

In all three trials, the plasma volume fell by ~9% at the end of the 75 min cycling, returned to baseline values during the 5 h recovery period before decreasing again during pre-load cycling and a 20 km time trial (*p* < 0.001; Figure 2). No differences were found among trials (*p* = 0.27).

### 3.9. Blood Glucose

Blood glucose at 1 h of recovery was higher with SD than WA (WA: 5.0 ± 0.3 mmol·L^−1^ vs. SD: 6.0 ± 0.7 mmol·L^−1^; *p* = 0.013) and with DD than WA at 2 h of recovery (WA: 5.0 ± 0.3 mmol·L^−1^ vs. DD: 6.1 ± 0.8 mmol·L^−1^; *p* = 0.005). Higher blood glucose was also found with SD and DD than with WA at the end of 45 min pre-load exercise (WA: 5.0 ± 0.3 mmol·L^−1^ vs. SD: 5.7 ± 0.4 mmol·L^−1^ vs. DD: 5.4 ± 0.4 mmol·L^−1^; *p* = 0.001). Likewise, higher blood glucose was observed with SD (5.8 ± 0.8 mmol·L^−1^; *p* = 0.001) and DD (5.5 ± 0.7 mmol·L^−1^; *p* = 0.01) than WA (4.6 ± 0.7 mmol·L^−1^) at the completion of the 20 km time trial.

### 3.10. Serum Osmolality and Electrolytes

**Figure 3:**
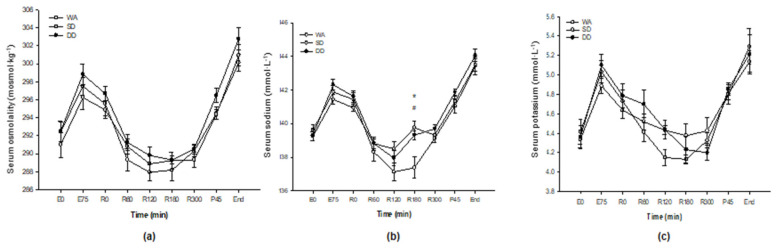
(**a**) Serum osmolality; (**b**) serum sodium concentration; and (**c**) serum potassium concentration. First bout of 75 min cycle: E0 and E75; 5 h recovery: R0–R300; end of pre-load: P45; completion of 20 km time trial: end. Values are the mean ± SE. * SD significantly different from WA (*p* < 0.05). ^#^ DD significantly different from WA (*p* < 0.05).

In all three trials, serum osmolality increased during the 75 min cycle, returned to baseline during recovery before increasing again during the pre-load cycle ride and 20 km time trial (*p* < 0.001; Figure 3a) but did not differ over time among trials (*p* = 0.60). Serum sodium concentration increased from the start to the end of the 75 min cycle in all three trials before falling during the 5 h recovery period (*p* < 0.001; Figure 3b). However, the fall in serum sodium was greater in the WA than SD and DD with a significant difference at 3 h of recovery (WA vs. SD, *p* = 0.010; WA vs. DD, *p* = 0.005). Serum potassium concentrations followed a similar response over time to sodium with an increase during all exercise bouts, but a decrease to baseline concentrations during the 5 h recovery period (*p* < 0.001; Figure 3c). No differences were found in the serum potassium over time among trials (*p* = 0.19).

### 3.11. Aldosterone and Anti-Diuretic Hormone

**Figure 4:**
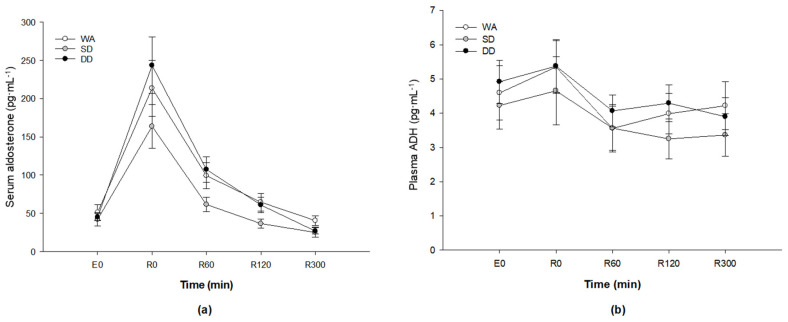
(**a**) Serum aldosterone concentration; and (**b**) plasma ADH concentration. Baseline: E0; 5 h recovery: R0–R300. Values are the mean ± SE.

Compared with the rest, serum aldosterone concentrations increased sharply during the 75 min cycling by about four-fold (317%) with WA, about four-fold (228%) with SD and about five-fold (441%) with DD before falling back toward baseline during the 5 h recovery period (*p* < 0.001; Figure 4a). There was no difference in the serum aldosterone among trials (*p* = 0.31). No differences were found in the plasma ADH concentrations among all three trials (*p* = 0.88; Figure 4b).

### 3.12. Twenty-Kilometre Time Trial Performance

**Figure 5:**
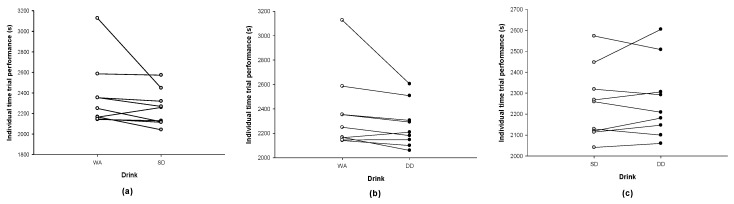
Trial against trial comparisons of the completion time for (**a**) WA and SD, (**b**) WA and DD, and (**c**) SD and DD.

Time trial performance was similar across trials (WA: 2365 ± 321 s vs. SD: 2252 ± 174 s vs. DD: 2268 ± 184 s; *p* = 0.65). In terms of individual results, the completion time was faster in eight participants with SD (Figure 5a) and seven participants with DD (Figure 5b) than with WA. Comparing SD with DD, the completion time was faster in five participants with SD and quicker with DD in four participants (Figure 5c). A small effect size was found between the WA and SD (0.42 (−0.47, 1.31)), and WA and DD (0.36 (−0.53, 1.24)) while the effect size was trivial between SD and DD (0.08 (−0.80, 0.96)).

## 4. Discussion

The primary aim of the present study was to examine the efficacy of ingesting an oral rehydration solution (denoted as DD) in restoring fluid balance during recovery compared with a commercially available sports drink (SD) and water (WA), after moderate–high intensity cycling exercise. Findings revealed a higher fluid retention in participants ingesting DD compared with ingesting WA or SD. Moreover, although DD and SD were rated as saltier than WA, this did not compromise the palatability of either drink. The second aim of this study was to examine the functional performance at the end of the recovery with the three drinks, with the volunteers completing a pre-load 20 km cycling time trial. Although the time trial performance was similar across trials, completion time was faster in eight out of nine participants with SD and seven out of nine participants with DD than with WA. Comparing SD with DD, the completion time was reduced in five participants and increased in four participants. The study design mimicked the lifestyle of elite endurance athletes and soldiers where two bouts of exercises can often be completed within a single day.

The two main factors influencing rehydration are the volume and the composition of fluid ingested [32]. In this study, the provision of isovolumetric solutions isolates the efficacy of each test solution to solely its constituents. The rationale to replenish with a volume that approximates 150% of sweat loss is to achieve a full restoration of body fluids after a preceding bout of exercise due to the obligatory sweat and urine losses that continue after exercise [14,33]. Similar sweat losses during the first 75 min exercise bout and total fluid intake among trials infer that the lower urine output during recovery is an effect of ingesting DD. The ingestion of DD achieved 34% greater retention of fluids than WA. This was likely due to the dilution of blood with water as shown by a decrease in serum sodium concentration, stimulating more urine losses [34]. Mean cumulative urine output was 424 mL lower with the ingestion of DD than with WA and ~358 mL lower (~20% greater retention of fluids) than with the SD.

To achieve the effective restoration of body fluids after exercise in the heat, it has been suggested that a drink should contain a sodium concentration of at least 50 mmol·L^−1^ [17]. In the present study, increased fluid retention was observed after consuming DD (with a sodium concentration of 60 mmol·L^−1^) compared with the SD (with a sodium concentration of 31 mmol·L^−1^) or WA. This observation is consistent with other published reports examining the influence of sodium concentrations in drinks on post-exercise fluid retention [10,35]. Compared with the ingestion of WA, Vrijens and Rehrer (1999) [36] reported higher plasma sodium concentrations in subjects exercising for 3 h in the heat when they ingested a carbohydrate–electrolyte beverage containing 18 mmol·L^−1^ of sodium. When this same solution (DD) was compared with a commercially available sports drink (containing 20 mmol·L^−1^ of sodium), fluid retention was found to be similar after 90 min of walking at 50% VO_2_max in a hot and dry environment while wearing wildland firefighter personal protective clothing [18]. However, it is noteworthy that the 30 min duration catered in that study for post-exercise rehydration assessment might not be sufficient to allow an accurate assessment of fluid retention. It is well established that two to six hours are required for solutions to be emptied and absorbed following ingestion [37]. In our study, a 5 h recovery period was allocated. Taken together, the present study supports these observations, as the serum sodium concentration was greater during recovery with the ingestion of DD and SD than WA although importantly, only in the case of DD did this higher sodium concentration translate into greater fluid retention during recovery.

Similar to other studies [9,35,38], a negative net fluid balance was present under all conditions at the end of the 5 h recovery period in the current study. However, higher fluid retention was achieved with DD than WA and this is was likely due to the dilution in serum sodium concentration with water, which stimulated an increased urine production [34]. Moreover, it is suggested that the drive to drink is reduced when fluids which contain a low sodium concentration are ingested. Thus, if the present study had examined ad libitum drinking on post-exercise fluid restoration, differences in intake between WA and DD may have been greater, further exacerbating the difference in net fluid balance.

The main reason for the inclusion of sodium in the formulation of rehydration beverages was because sodium is the primary ion lost in sweat. It is also well established that net fluid retention is related to the concentration of electrolytes, mainly sodium, in the solution [9,14,39]. During recovery, the inclusion of sodium offers several advantages over plain water: (i) it sustains the osmotic drive to drink [34] thereby promoting better voluntary fluid intake [40]; (ii) maintains greater plasma and extracellular fluid volumes [41]; (iii) lowers urine output; and (iv) blunts further declines in plasma sodium concentration [36]. In addition to stimulating greater urine losses caused by the dilution of blood, a decline in plasma sodium concentration also presents a higher risk of developing hyponatraemia which is defined as a serum or plasma sodium concentration <135 mmol/L [42] and this can occur during exercise or in the post-exercise period. This condition has been observed in exercising individuals consuming large volumes of fluid during exercise or who replace sweat losses with fluids containing no/low sodium concentration [3,43]. We observed higher serum sodium concentration with the ingestion of DD and SD than with WA during recovery while similar serum sodium concentration was found between DD and SD. This implies that DD may better restore serum sodium and potentially minimise EAH during subsequent exercise bouts on the same day than WA but is comparable with SD. However, EAH cases are usually found in prolonged activities (e.g., marathons or ultra-endurance activities) which the shorter exercise duration of 75 min in this study is not representative.

While it seems obvious to increase the sodium concentration during the formulation of rehydration beverages, high sodium content is reported to decrease the palatability of drinks [39,44]. Despite the high sodium content (60 mmol·L^−1^) in DD, our findings revealed no difference in the ratings of sweetness, saltiness, palatability and mouth taste between DD and SD. Higher mouth taste refers to better after-taste following the ingestion of drink. Moreover, in terms of palatability, both DD and the SD were similar to WA but were considered to be more thirst quenching and to have a better mouth taste.

It has been documented that aldosterone regulates the concentrations of sodium and potassium, and plays an important role in sodium homeostasis. In this study, an increase in serum aldosterone concentration was found at the end of the 75 min cycling, accompanied by an increase in serum sodium and potassium concentrations. This observation is similar to other studies [13,16]. Nose et al. [16] found an increase in plasma sodium, potassium and aldosterone concentrations after six male participants exercised in the heat to induce 2.3% dehydration. They also found that an increase in urinary loss of sodium and water was associated with a reduction in the stimulus for aldosterone release. In the present study, similar serum aldosterone concentration was found among trials, despite a lower cumulative urine output and higher serum sodium concentration being observed during recovery with the ingestion of DD than WA. This may be due to the higher sodium and potassium concentrations in DD which increased the activity of the peritubular sodium/potassium pump, i.e., the reabsorption of sodium will increase with increasing excretion of potassium [5]. However, urinary sodium and potassium concentrations were not analysed so this cannot be confirmed.

ADH is released to increase the water permeability of kidney collecting tubules, decreasing urine flow and increasing net water re-absorption [12]. Plasma volume and osmolality are the main factors involved in the regulation of plasma ADH concentrations [45,46] and a decrease in plasma volume and an increase in serum osmolality were observed after the 75 min cycle in all conditions. However, unlike previous studies [13,47], no increase in plasma ADH concentrations was seen after exercise in this study. A possible reason may be that the comparison was made with plasma volume and serum osmolality at the end of the 75 min cycle and plasma ADH concentrations at 0 h of recovery. It has been shown that the half-life of ADH clearance from blood circulation is between 10 and 20 min [12,48]. Our protocol included a period of towel drying and nude body mass measurement, meaning that the delay from the end of the 75 min cycle to the start of recovery was about 12 min, which may explain the absence of the influence of plasma volume and serum osmolality on plasma ADH concentrations after exercise-induced dehydration. An alternative explanation is that the regular feeding of fluid boluses during exercise in the present investigation blunted changes in plasma volume and osmolality and subsequently ADH. Certainly, similar changes were observed in these variables at the end of the exercise.

It is important to replace fluid deficits after a preceding bout of activity since it is accepted that any deficit incurred prior to the next bout of exercise could lead to impairment in endurance performance [49,50]. In the present study, no differences in endurance performance were found among the test drinks which is consistent with the studies conducted in field settings where the efficacy of sodium supplementation was evaluated [51,52]. It is noteworthy that a standardised breakfast was provided in the present investigation, to ensure that the participants did not commence the trials with any caloric deficit from overnight fasting. Moreover, the breakfast would have replenished any liver glycogen stores depleted by an overnight fast. This implies that the importance of substrate provision during the experiments might have been lower as compared with previous studies where participants were fasted overnight [7,53]. Another possible reason may be that the participants were still in negative fluid balance at the end of recovery and they commenced the time trial in fluid deficit. Nevertheless, there was a trend towards better performance with SD and DD as compared with WA in our study. When comparing between the SD and DD, similar performance was observed with an ~50% equal distribution in performance time. Thus, it seems more likely that DD and SD have similar effects on endurance performance in the heat.

Given the relatively small sample size, the possibility of a type 2 error should not be ignored in experimental research such as this. Effect sizes were small in comparing the time trial completion time among drinks. However, small effect sizes for both the SD vs. WA (WA vs. SD: 0.42 (−0.47, 1.31)) and DD vs. WA (WA vs. DD: 0.36 (−0.53, 1.24)) should not be discounted given that in competitive races, a few seconds’ difference in finishing time may differentiate the winner from the first runner-up. For instance, in the Women’s Elite category of the OCBC Cycle National Road Championships 2019 held in Singapore, the differences in timing between the winner (02:37:29.60), the first runner-up (02:37:29.73) and the second runner-up (02:37:30.02) who completed the race were less than 1 s [54]. Furthermore, in the Men’s Elite category, the difference in finishing time between the first runner-up (03:02:20.56) and the second runner-up was also less than 1 s (03:02:20.64) [54]. Teasing out such effects in experimental studies, such as the present one, where participant numbers are often small and where results are measured in seconds or less is difficult. Moreover, such differences are often confounded by issues such as motivation in experimental settings. Thus, collective information from future meta-analyses might better answer the question than small, single experimental studies in isolation.

## 5. Conclusions

In conclusion, the ingestion of DD resulted in lower cumulative urine output and a higher fluid retention than SD and WA after the endurance exercise in a hot and humid environment. The ingestion of a SD, however, led to a greater fluid retention than WA. Thus, for individuals who exercised in the heat and who performed multiple exercise sessions each day, DD represented a potential rehydration beverage that they could turn to help maintain better fluid balance. Although no differences were found in the time trial performance among the drinks, a small effect size for better performance with SD and DD compared with WA was observed.

## Figures and Tables

**Table 1:** The energy content and composition of test drinks. Energy content and macronutrients concentration are obtained from the manufacturer and are presented as the mean. Osmolality and the concentration of electrolytes are the mean ± SD. Tap water is not included for analysis.

Drink Contents	Sports Drink (SD)	Oral Rehydration Solution (DD)
Energy value (kcal·L^−1^)	250	130
Fat (g·L^−1^)	0	0
Protein (g·L^−1^)	0	0
Carbohydrate (g·L^−1^)	62	33
Osmolality (mOsmol·kg^−1^)	382 ± 31	216 ± 4
Sodium (mmol·L^−1^)	31 ± 3	60 ± 3
Potassium (mmol·L^−1^)	5.3 ± 0.3	18.2 ± 0.4

**Table 2:** Pre-trial physiological parameters.

Parameter	Water (WA)	Sports Drink (SD)	Oral Rehydration Solution (DD)	*P*_ANOVA_
Body mass (kg)	63.90 ± 7.61	63.94 ± 7.38	63.38 ± 7.54	0.98
Haemoglobin (g·L^−1^)	137 ± 10	138 ± 7	139 ± 10	0.92
Haematocrit (%)	42.6 ± 2.7	42.9 ± 2.2	42.8 ± 2.7	0.96
Urine osmolality (mOsmol·kg^−1)^	683 ± 344	572 ± 269	606 ± 309	0.74
Serum osmolality (mOsmol·kg^−1^)	292 ± 3	291 ± 4	293 ± 3	0.59
Serum sodium (mmol·L^−1^)	140 ± 1	139 ± 1	139 ± 1	0.50
Blood glucose (mmol·L^−1^)	5.6 ± 1.0	5.7 ± 1.1	5.7 ± 0.8	0.94
Serum potassium (mmol·L^−1^)	4.3 ± 0.3	4.4 ± 0.4	4.4 ± 0.3	0.86
Core temperature (°C)	36.9 ± 0.2	36.9 ± 0.2	37.0 ± 0.1	0.46
Heart rate (b·min^−1^)	67 ± 10	69 ± 10	66 ± 7	0.63

## References

[1] Maughan R. (2003). Impact of mild dehydration on wellness and on exercise performance. Eur. J. Clin. Nutr..

[2] Sawka M.N. (1992). Physiological consequences of hypohydration: Exercise performance and thermoregulation. Med. Sci. Sports Exerc..

[3] Hew-Butler T., Almond C., Ayus J.C., Dugas J., Meeuwisse W., Noakes T., Reid S., Siegel A., Speedy D., Stuempfle K. (2005). Consensus statement of the 1st international exercise-associated hyponatremia consensus development conference, Cape Town, South Africa 2005. Clin. J. Sport Med..

[4] Noakes T., Sharwood K., Speedy D., Hew T., Reid S., Dugas J., Almond C., Wharam P., Weschler L. (2005). Three independent biological mechanisms cause exercise-associated hyponatremia: Evidence from 2,135 weighed competitive athletic performances. Proc. Natl. Acad. Sci..

[5] Maughan R., Murray R. (2000). Sports Drinks: Basic Science and Practical Aspects.

[6] Gonzalez-Alonso J., Heaps C., Coyle E. (1992). Rehydration after exercise with common beverages and water. Int. J. Sports Med..

[7] Lee J.K., Nio A.Q., Ang W.H., Law L.Y., Lim C.L. (2011). Effects of ingesting a sports drink during exercise and recovery on subsequent endurance capacity. Eur. J. Sport Sci..

[8] Nielsen B., SjØgaard G., Ugelvig J., Knudsen B., Dohlmann B. (1986). Fluid balance in exercise dehydration and rehydration with different glucose-electrolyte drinks. Eur. J. Appl. Physiol. Occup. Physiol..

[9] Maughan R., Leiper J. (1995). Sodium intake and post-exercise rehydration in man. Eur. J. Appl. Physiol. Occup. Physiol..

[10] Shirreffs S.M., Maughan R.J. (1998). Volume repletion after exercise-induced volume depletion in humans: Replacement of water and sodium losses. Am. J. Physiol. Ren. Physiol..

[11] Maughan R.J., Murray R. (2001). Fundamentals of sports nutrition: Application to sports drink. Sports Drinks: Basic Science and Practical Aspects.

[12] Verbalis J.G. (2003). Disorders of body water homeostasis. Best Pract. Res. Clin. Endocrinol. Metab..

[13] Montain S.J., Laird J.E., Latzka W.A., Sawka M.N. (1997). Aldosterone and vasopressin responses in the heat: Hydration level and exercise intensity effects. Med. Sci. Sports Exerc..

[14] Shirreffs S.M., Taylor A.J., Leiper J.B., Maughan R.J. (1996). Post-exercise rehydration in man: Effects of volume consumed and drink sodium content. Med. Sci. Sports Exerc..

[15] Horton R. (1973). Aldosterone: Review of its physiology and diagnostic aspects of primary aldosteronism. Metabolism.

[16] Nose H., Mack G.W., Shi X., Nadel E.R. (1988). Involvement of sodium retention hormones during rehydration in humans. J. Appl. Physiol..

[17] Maughan R., Shirreffs S. (1997). Recovery from prolonged exercise: Restoration of water and electrolyte balance. J. Sports Sci..

[18] Schleh M.W., Dumke C.L. (2018). Comparison of sports drink versus oral rehydration solution during exercise in the heat. Wilderness Environ. Med..

[19] Maughan R.J., Watson P., Cordery P.A., Walsh N.P., Oliver S.J., Dolci A., Rodriguez-Sanchez N., Galloway S.D. (2016). A randomized trial to assess the potential of different beverages to affect hydration status: Development of a beverage hydration index. Am. J. Clin. Nutr..

[20] DuBois D. (1916). A formula to estimate the approximate surface area if height and body mass be known. Arch. Intern. Med..

[21] Durnin J.V., Womersley J. (1974). Body fat assessed from total body density and its estimation from skinfold thickness: Measurements on 481 men and women aged from 16 to 72 years. Br. J. Nutr..

[22] Siri W.E. (1956). The gross composition of the body. Advances in Biological and Medical Physics.

[23] Borg G. (1973). Perceived exertion: A note on" history" and methods. Med. Sci. Sports.

[24] Reilly T., Brooks G. (1986). Exercise and the circadian variation in body temperature measures. Int. J. Sports Med..

[25] Forster H., Dempsey J., Thomson J., Vidruk E., DoPico G. (1972). Estimation of arterial PO2, PCO2, pH, and lactate from arterialized venous blood. J. Appl. Physiol..

[26] Young A.J., Sawka M.N., Epstein Y., Decristofano B., Pandolf K.B. (1987). Cooling different body surfaces during upper and lower body exercise. J. Appl. Physiol..

[27] Romer L.M., Bridge M.W., McConnell A.K., Jones D.A. (2004). Influence of environmental temperature on exercise-induced inspiratory muscle fatigue. Eur. J. Appl. Physiol..

[28] Sewell D.A., McGregor R.A. (2008). Evaluation of a cycling pre-load time trial protocol in recreationally active humans. Eur. J. Appl. Physiol..

[29] Thompson W.O., Thompson P.K., Dailey M.E. (1928). The effect of posture upon the composition and volume of the blood in man. J. Clin. Investig..

[30] Dill D.B., Costill D.L. (1974). Calculation of percentage changes in volumes of blood, plasma, and red cells in dehydration. J. Appl. Physiol..

[31] Hopkins W., Marshall S., Batterham A., Hanin J. (2009). Progressive statistics for studies in sports medicine and exercise science. Med. Sci. Sports Exerc..

[32] Shirreffs S.M., Armstrong L.E., Cheuvront S.N. (2004). Fluid and electrolyte needs for preparation and recovery from training and competition. J. Sports Sci..

[33] Mitchell J.B., Grandjean P.W., Pizza F.X., Starling R.D., Holtz R.W. (1994). The effect of volume ingested on rehydration and gastric emptying following exercise-induced dehydration. Med. Sci. Sports Exerc..

[34] Nose H., Mack G.W., Shi X., Nadel E.R. (1988). Role of osmolality and plasma volume during rehydration in humans. J. Appl. Physiol..

[35] Merson S.J., Maughan R.J., Shirreffs S.M. (2008). Rehydration with drinks differing in sodium concentration and recovery from moderate exercise-induced hypohydration in man. Eur. J. Appl. Physiol..

[36] Vrijens D., Rehrer N. (1999). Sodium-free fluid ingestion decreases plasma sodium during exercise in the heat. J. Appl. Physiol..

[37] Leiper J.B., Maughan R., Murray R. (2001). Gastric Emptying and Intestinal Absorption of Fluids, Carbohydrates, and Electrolytes.

[38] Shirreffs S.M., Aragon-Vargas L.F., Keil M., Love T.D., Phillips S. (2007). Rehydration after exercise in the heat: A comparison of 4 commonly used drinks. Int. J. Sport Nutr. Exerc. Metab..

[39] Wemple R.D., Morocco T.S., Mack G.W. (1997). Influence of sodium replacement on fluid ingestion following exercise-induced dehydration. Int. J. Sport Nutr. Exerc. Metab..

[40] Passe D.H. (2001). Physiological and Psychological Determinants of Fluid Intake. Sports Drinks: Basic Science and Practical Aspects.

[41] Below P.R., Mora-Rodriguez R., Gonzalez-Alonso J., Coyle E.F. (1995). Fluid and carbohydrate ingestion independently improve performance during 1 h of intense exercise. Med. Sci. Sports Exerc..

[42] Kratz A., Lewandrowski K.B., Siegel A.J., Chun K.Y., Flood J.G., Van Cott E.M., Lee-Lewandrowski E. (2002). Effect of marathon running on hematologic and biochemical laboratory parameters, including cardiac markers. Am. J. Clin. Pathol..

[43] Davis D.P., Videen J.S., Marino A., Vilke G.M., Dunford J.V., Van Camp S.P., Maharam L.G. (2001). Exercise-associated hyponatremia in marathon runners: A two-year experience. J. Emerg. Med..

[44] Nadel E.R. (1990). Influence of Fluid-Replacement Beverages on Body Fluid Homeostasis during Exercise and Recovery. Perspect. Exerc. Sci. Sports Med..

[45] Brandenberger G., Candas V., Follenius M., Kahn J. (1989). The influence of the initial state of hydration on endocrine responses to exercise in the heat. Eur. J. Appl. Physiol. Occup. Physiol..

[46] Wade C.E. (1984). Response, regulation, and actions of vasopressin during exercise: A review. Med. Sci. Sports Exerc..

[47] Melin B., Jimenez C., Savourey G., Bittel J., Cottet-Emard J., Pequignot J., Allevard A., Gharib C. (1997). Effects of hydration state on hormonal and renal responses during moderate exercise in the heat. Eur. J. Appl. Physiol. Occup. Physiol..

[48] Coyle E.F., Coggan A.R. (1984). Effectiveness of carbohydrate feeding in delaying fatigue during prolonged exercise. Sports Med..

[49] Maughan R. (1991). Fluid and electrolyte loss and replacement in exercise. J. Sports Sci..

[50] Maughan R. (1991). Effects of CHO-electrolyte solution on prolonged exercise. Perspect. Exerc. Sci. Sports Med..

[51] Hew-Butler T., Sharwood K., Collins M., Speedy D., Noakes T. (2006). Sodium supplementation is not required to maintain serum sodium concentrations during an Ironman triathlon. Br. J. Sports Med..

[52] Speedy D.B., Thompson J.M., Rodgers I., Collins M., Sharwood K. (2002). Oral salt supplementation during ultradistance exercise. Clin. J. Sport Med..

[53] Maughan R., Fenn C., Leiper J. (1989). Effects of fluid, electrolyte and substrate ingestion on endurance capacity. Eur. J. Appl. Physiol. Occup. Physiol..

[54] Cycosports OCBC Cycle National Road Championships 2019-Lap Result List. https://my1.raceresult.com/127625/RRPublish/pdf.php?name=Result%20Lists%7CLap%20Result%20List&contest=0&lang=en.

