# Correction: Fan et al. Efficacy of Ingesting an Oral Rehydration Solution after Exercise on Fluid Balance and Endurance Performance. *Nutrients* 2020, *12*, 3826

**DOI:** 10.3390/nu13113855

**Published:** 2021-10-28

**Authors:** Priscilla Weiping Fan, Stephen F. Burns, Jason Kai Wei Lee

**Affiliations:** 1 DSO National Laboratories, Defence Medical and Environmental Research Institute, Singapore S117510, Singapore; fweiping@dso.org.sg; 2 Physical Education and Sports Science, National Institute of Education, Nanyang Technological University, Singapore S637616, Singapore; stephen.burns@nie.edu.sg; 3 Human Potential Translational Research Programme, Yong Loo Lin School of Medicine, National University of Singapore, Singapore S117597, Singapore; 4 Department of Physiology, Yong Loo Lin School of Medicine, National University of Singapore, Singapore S117593, Singapore; 5 Global Asia Institute, National University of Singapore, Singapore S119076, Singapore; 6 N.1 Institute for Health, National University of Singapore, Singapore S117456, Singapore; 7 Institute for Digital Medicine, National University of Singapore, Singapore S117456, Singapore

The authors would like to make a correction in a recent published paper [1]. There was an error in Figure 1b. In the original Figure 1b, the black line representing the mean of each datum was labeled incorrectly.

Original Figure 1b:


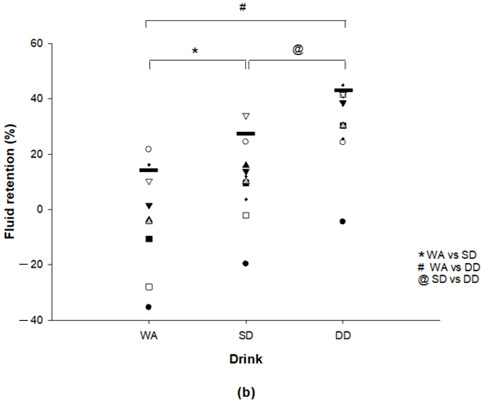


We would like it to be corrected as shown below.

New Figure 1b:


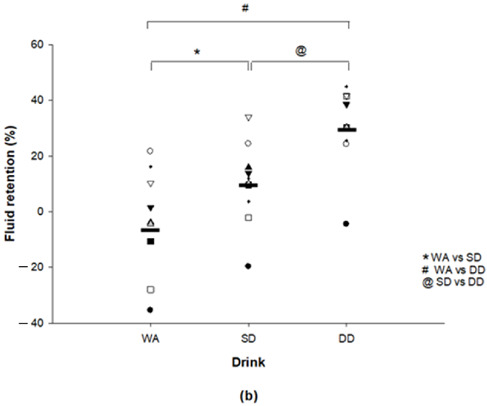


These changes have no material impact on the conclusions of the paper. The authors would like to apologize to the readers of *Nutrients* for this error. The published version will be updated on the article webpage, with a reference to this correction notice.
